# Expression of salivary hepcidin and its inducer, interleukin 6 as well as type I interferons are significantly elevated in infants with poor oral rotavirus vaccine take in South Africa

**DOI:** 10.3389/fimmu.2025.1517893

**Published:** 2025-05-16

**Authors:** Vusumuzi Mabasa, Mapeseka L. Seheri, Cliff A. Magwira

**Affiliations:** Diarrheal Research Pathogens Unit (DPRU), Department of Virology, School of Medicine, Sefako Makgatho Health Sciences University, Pretoria, South Africa

**Keywords:** iron deficiency anemia, hepcidin, IL-6, interferons, rotavirus vaccine, shedding

## Abstract

**Background:**

The metabolism of nutritional iron, required by viruses to replicate and proliferate, is regulated by hepcidin, a peptide hormone of 25 amino acids. Overexpression of hepcidin results in iron deficiency anemia (IDA), while its downregulation can lead to an iron overload. Recently, IDA has been highlighted for its role in the impairment of adaptive immunity and poor vaccine effectiveness. Here, we assessed the possible role of IDA in oral rotavirus (RV) vaccine take among South African infants.

**Methodology:**

Paired stool and unstimulated saliva were collected from oral RV vaccinated infants, who attended a routine immunization program at Oukasie Healthcare clinic, north of Pretoria, South Africa, to decide vaccine shedders (n = 20) and non-shedders (n = 18). IDA was determined by assaying the salivary hepcidin levels using an ELISA kit, while expression of hepcidin, hepcidin inducer IL-6, Interferon I (IFN I) and IFN-γ were determined by qPCR.

**Results:**

There were no significant differences in the average hepcidin levels between vaccine shedders and non-shedders (*p* = 0.83). Hepcidin levels were decreased 0.43-fold a week after vaccination versus pre-vaccination, *p* = 0.0001. Unlike the concentration, the expression of hepcidin increased 5-5-fold in non-shedders compared to vaccine shedders. Similarly, the expression of IL-6 and IFN I were increased 5.2- and 4.9-fold, respectively, in non-shedders compared to shedders. In contrast, the expression of IFN-γ was increased 3.2-fold in shedders compared to non-shedders.

**Summary:**

Collectively, our observations suggest a possible role for IDA in impediment of oral RV vaccine take among South African infants.

## Background

Rotavirus (RV) gastroenteritis remains one of the leading causes of childhood morbidity and mortality in children under the age of five in low-middle income countries (LMICs) (1). Vaccines for preventing severe RV gastroenteritis are available but perform poorly in LMICs (2, 3) due to a variety of factors. Recently, malnutrition, especially iron deficiency anemia (IDA), has been highlighted for its role in the impairment of adaptive immunity and vaccine effectiveness (4).

Iron is fundamental to many biological processes including DNA/RNA synthesis and ATP generation (5). Its absorption from the intestines and distribution to tissues is regulated by hepcidin (6), a peptide hormone of 25 amino acids (7). Its binding to ferroportin internalizes and degrades the protein, thereby preventing the release of iron from cells (8). Hepcidin expression is regulated by a multiple of stimuli including serum iron levels, inflammations and infections (9). The overexpression of hepcidin results in iron deficiency anemia, while its downregulation can lead to an iron overload (10). Viruses depend on the host cells for their replication (11), and requires iron replete cells to successfully propagate themselves. As such, iron bioavailability or overload can lead to an increase in viral replication and viral load, and its deficiency in ineffective viral infection. Since iron is essential to viral replication, viruses have developed mechanisms to ensure that the infected cells are iron replete including regulation of hepcidin expression (12).

IDA can present as absolute IDA, in which the body stores and bone marrow ion availability is not adequate, or functional IDA, in which the total body iron stores are sufficient but unavailable in the circulation to support erythropoiesis due to increased levels of hepcidin. IDA at the time of vaccination has recently been shown to be predictive of decreased response to diphtheria, pertussis and pneumococcal vaccines, while its supplementation prior to immunization increased the response to these vaccines (13). We hypothesize that infants with high hepcidin levels (IDA positive) respond poorly to oral RV vaccination. Here, we report on differences in salivary hepcidin and its inducer inflammatory cytokine IL-6 expression between RV vaccine responders and non-responders. We also report on differences in type I and III interferon (IFN) responses between the two study groups following oral Rotarix vaccination.

## Materials and methods

### Study design, subjects and specimen collection

This cross-sectional study involved the use of paired stool and unstimulated saliva collected from infants who reported at Oukasie Healthcare clinic in Oukasie, Brits, north of Pretoria, for routine Rotarix (GlaxoSmithKline Biologicals, Belgium) immunization. Oukasie is a predominantly poor black township with significant socioeconomic challenges. Saliva and stool specimens were collected before vaccination on the day of the first dose (6 weeks) and 7 days post-vaccination (7DPV). Only healthy (physical) infants of similar age and weight (>2 kg) were enrolled for the study after written and informed consent from parents. Study infants were randomly selected and divided into two groups: those who shed the vaccine virus in stool samples (vaccine shedders) and those who did not shed the virus (non-shedders). The study protocol was approved by Sefako Makgatho Health Sciences University Research and Ethics Committee (SMUREC), number SMUREC/M/241/2021: PG.

Stool samples were collected from the infants’ diapers into sterile plastic bottles and immediately stored in −20°C freezers located at the clinic. Unstimulated saliva samples were collected by placing oral swabs in the mouth for 1 minute and rubbing them against the cheeks before putting them in sterile 15 mL plastic tubes and kept frozen at −20°C. Frozen samples were transported to the laboratory in cooler boxes packed with ice blocks.

### Viral RNA extraction and detection of vaccine in stool samples

Viral RNA was extracted from stool samples using FavorPrep Viral RNA Mini Extraction Kit (FAVORGEN Biotech Corp, Vienna, Austria) following the manufacturer’s instructions. The RV vaccine was detected in viral RNA samples by reverse transcriptase polymerase chain reaction (RT-PCR) using Luna One-step RT-PCR kit (New England Biolabs, Massachusetts, USA) and NSP2 primers and a probe (14). The amplification was performed in Bio-Rad CFX96 Real-Time System (Bio-Rad Laboratories, Hercules, California) as described previously [Gautam et al., 2014].

### Salivary hepcidin level assay

IDA was determined by assaying the hepcidin levels in saliva specimens using human standardized Hepc25 ELISA kit (Elabscience, Houston, USA) following the manufacturer’s instructions. The kit has a detection limit of 1.56–100 ng/mL and intra-/inter-assay variability of coefficient of variation (CV)<8% and CV<10%, respectively. The iron status was captured as IDA positive if the hepcidin concentration level was above 5.5ng/ml (habitual ion blockers), and IDA negative if the hepcidin concentration levels were in the ranges of 1.56 ng/ml < Hepcidin < 5.5 ng/ml (habitual ion absorbers) as described by Prentice et al. (15).

### Total human RNA extraction and cDNA synthesis

Total human RNA was extracted from saliva samples using Isolate II RNA Mini Kit (Bioline Meridian Bioscience, United Kingdom) as described by the manufacturer. The extracted RNA was converted to complementary DNA (cDNA) using Tetro cDNA Synthesis kit (Bioline Meridian Bioscience, United Kingdom) in a GeneAmp PCR System 9700 (Applied Biosystems, Waltham, MA, USA) under the following conditions: incubation at 25°C for 10 minutes, followed by reverse transcription at 45°C for 40 minutes. The reaction was terminated by incubating at 85°C for 5 minutes and the cDNA stored at -20°C until further use.

### Expression of hepcidin, IL-6, IFN I and IFN-ƴ genes

The expression of genes coding for hepcidin (HAMP), interleukin 6 (IL-6), Myxovirus resistance A (MxA, a biomarker for secreted IFN-α/β), and IFN-ƴ was assayed by qPCR using 2X Luna qPCR kit (New England Biolabs, Massachusetts, USA) and primers: MxAF and MxAR (16); GAPGHF and GAPDHR (16); HEPCF1 and HEPCR1 (17); IFN-ƴ and IFN-ƴ R (18); IL-6F and IL-6R (19). Briefly, a 20 µl PCR reaction mixture consisted of 2X Luna qPCR kit (New England Biolabs, Massachusetts, USA), 0.4 µM each of forward and reverse primers, 5 µl of cDNA and sterile water. Amplification was performed in a CFX 96 Real-Time System (Bio-Rad Laboratories, Hercules, California) under the following conditions: initial denaturation of at 95°C for 2 minutes, followed by 45 cycles of denaturation at 95°C for 10 seconds, and extension at 60°C for 30 seconds, with a melt curve insertion (65°C to 95°C: Increment 0.5°C for 0:05 s). Gene expression was normalized with the housekeeping glyceraldehyde-3-phosphate dehydrogenase gene (GAPDH) amplification as described previously (20).

### Data and statistical analysis

All data analysis and data visualizations were done using R, version 2.2.4 (Rstudio, Boston, Massachusetts, USA). The frequency (proportion) of functional iron deficiency anemia was measured by dividing the number of infants positive for IDA by the total number of infants in a dataset. The distribution of hepcidin was measured using measures of spread (mean and standard deviation) and the coefficient of variation (CV), where the CV was used as a standardized measure of distribution. Fold-change gene expression was calculated using delta Ct as described by Schmittgen and Livak (20). An unpaired t test was used to measure the differences in mean gene expression between the two study groups. The generalized logistic regression was used to evaluate associations between the expressions of MxA, IFN-ƴ or hepcidin and rotavirus shedding. In all statistical tests, *p* ≤ 0.05 was regarded as statistically significant.

## Results

### Demographics

A total of 121 RV vaccine recipients were recruited and eligible for the study. The majority (75%) of infants were born naturally and the rest via C-section. Females made up most of the participants (58%) and 42% were males. Over half (55%) of the infants were fed with both breastmilk and formula while 45% were fed exclusively on breastmilk. There were no significant differences in demographics between vaccine shedders and non-shedders, except for feeding type (**Table 1**).

**Table 1:** Demographics and other characteristics of study infants.

Characteristic	Overall, n (%)	Non-shedders, n (%)	Shedders, n (%)	p-value^1^
Age				
07 weeks	121 (100%)	49 (40%)	72 (60%)	
**Sex**				0.91
Females	70 (58%)	28 (57%)	42 (58%)	
Males	51 (42%)	21 (43%)	30 (42%)	
Race				
Black	121 (100%)	49 (41%)	72 (59%)	
**Birth delivery**				0.40
C-section	30 (25%)	14 (29%)	16 (22%)	
NVD	91 (75%)	35 (71%)	56 (78%)	
**Feeding type**				0.02
Breastmilk	55 (45%)	16 (33%)	39 (54%)	
Breastmilk/Formula	66 (55%)	33 (67%)	33 (46%)	

^1^Pearson’s Chi-squared test.

### Frequency of IDA in study infants

A total of 48 saliva samples, 30 from vaccine shedders and the rest from non-shedders were randomly selected for hepcidin testing. Prior to vaccination, all (100%) of vaccine shedders and 95% of non-shedders had hepcidin concentration below <5.5ng/ml (i.e. were negative for IDA). Only 5% of non-shedders had hepcidin levels above 5.5ng/ml (positive for IDA). At 7DPV, all (100%) of both vaccine shedders and non-shedders were also IDA negative.

### Salivary hepcidin levels decrease after vaccination

**Figure 1:**
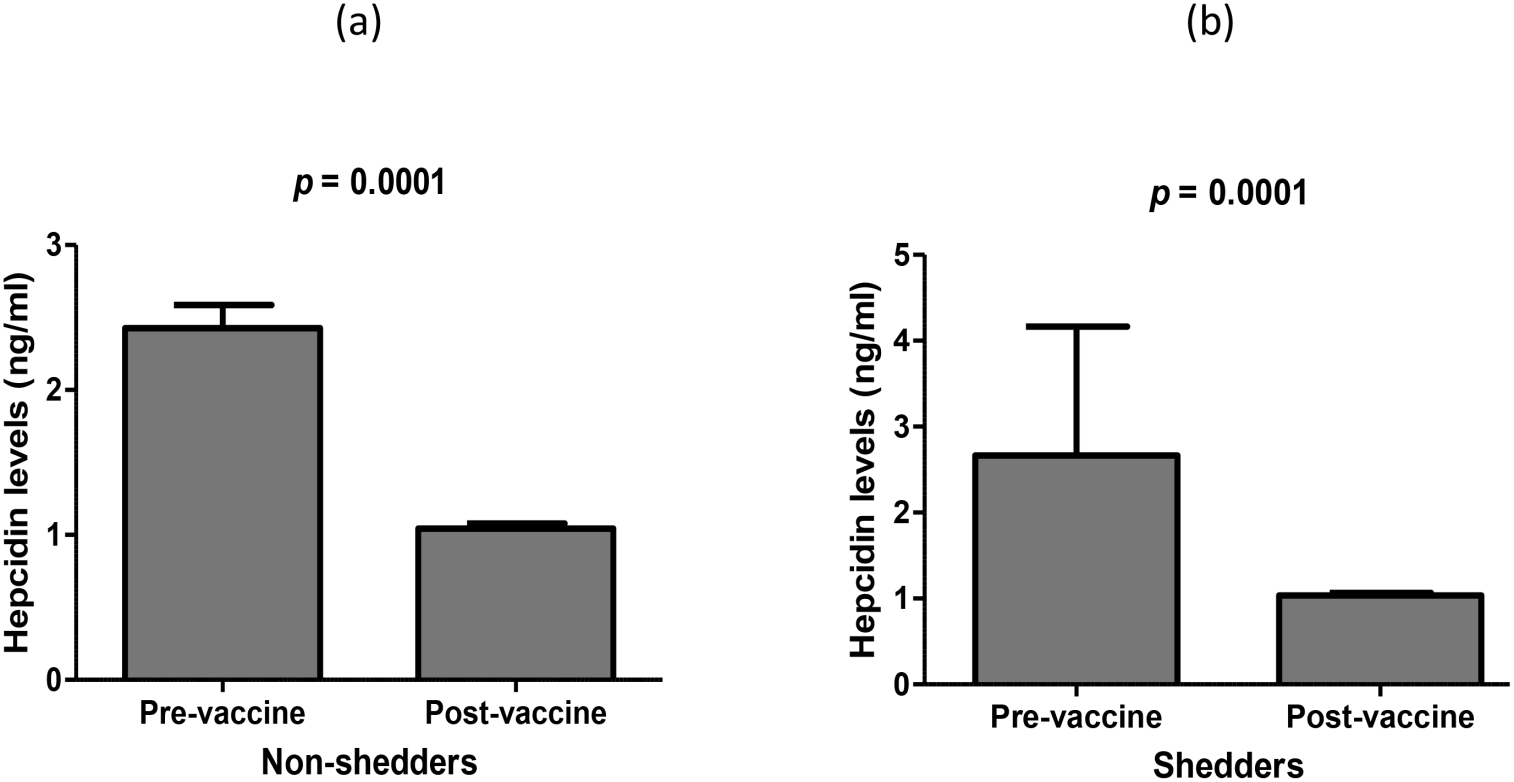
Average levels of salivary hepcidin prior to oral RV vaccination at week 6 versus 7 days post RV vaccination in **(a)** non-shedders and **(b)** vaccine shedders.

There were no significant differences in mean concentration of salivary hepcidin between vaccine shedders and non-shedders prior to vaccination, *p* = 0.46 (**Table 2**). Similarly, the hepcidin levels between vaccine shedders and non-shedders at 7DPV were not significantly different, *p* = 0.83 (**Table 2**). However, there was 0.43-fold drop in average hepcidin levels in vaccine shedders from pre-vaccination to 7DPV, *p* = 0.0001 (**Figure 1a**). Similarly, the hepcidin level decreased 0.4-fold in non-shedders from pre-vaccination to 7DPV level, *p* = 0.0001 (**Figure 1b**).

**Table 2:** Hepcidin levels in vaccine shedders versus non-shedders pre- and post-vaccination.

	Pre-vaccination		Post-vaccination	
Shedding status	Hepcidin (Mean ± SD)	CV (%)	Hepcidin (Mean ± SD)	CV (%)
Vaccine shedders	2.43 ± 0.16	6.60	1.04 ± 0.17	16.13
Non-shedders	2.67 ± 1.50	56.22	1.04 ± 0.14	13.70

### Hepcidin expression different between vaccine shedders and non-shedders

**Figure 2:**
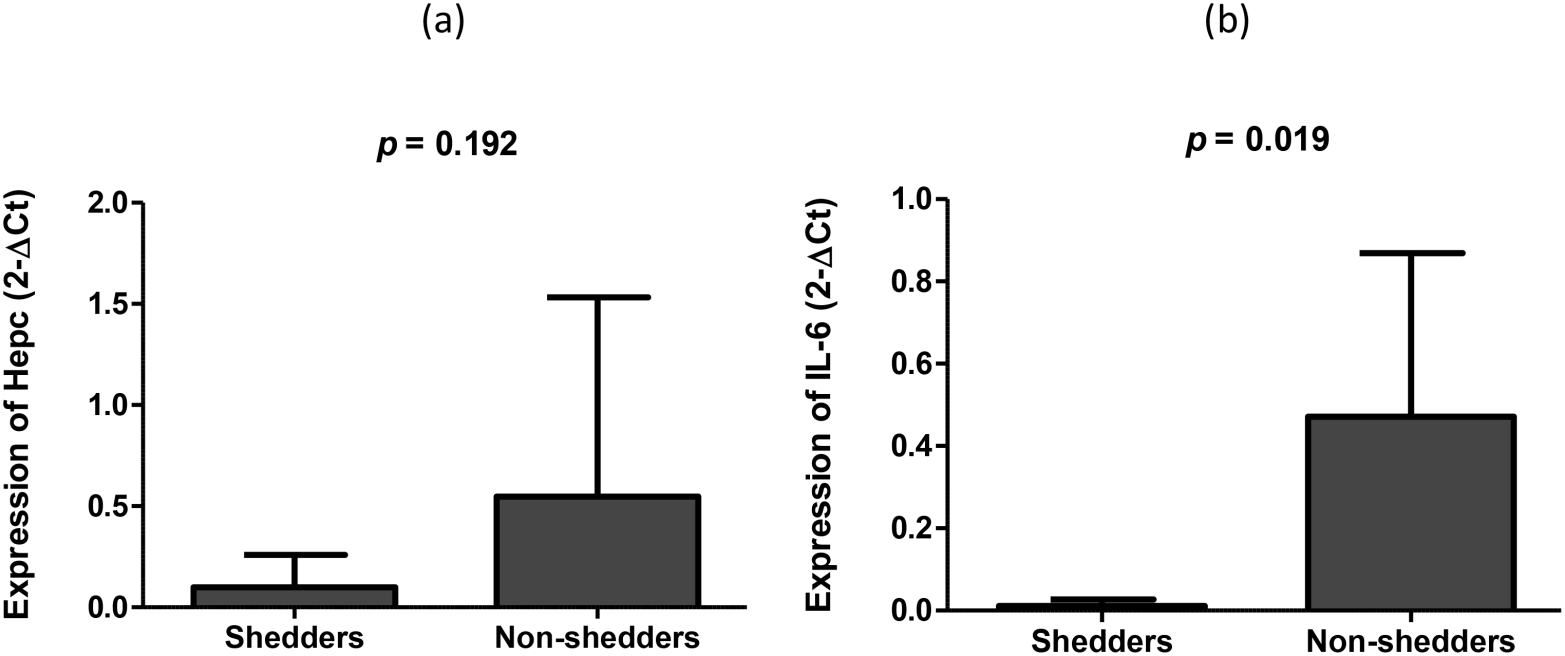
Average expression levels of **(a)** Hepcidin and **(b)** IL-6 in saliva mRNA samples of the two study populations 7 days after oral RV vaccination.

We tested whether the expression level of salivary hepcidin was consistent with concentration of salivary hepcidin between the two study groups. Unlike the concentration, the average expression of hepcidin was increased 5.5-fold in non-shedders (2-ΔCt = 0.548) versus shedders (2-ΔCt = 0.099) (**Figure 2a**). We also assessed whether the hepcidin expression in the two study groups corresponded with the expression of inflammatory hepcidin inducer, IL-6, and found that the expression of the cytokine was increased 5.2-fold in non-shedders (2-ΔCt = 0.471) compared to non-shedders (2-ΔCt = 0.091), *p* = 0.019 (**Figure 2b**).

### Significant difference in expression of MxA and IFN-γ between vaccine shedders and non-shedders

**Figure 3:**
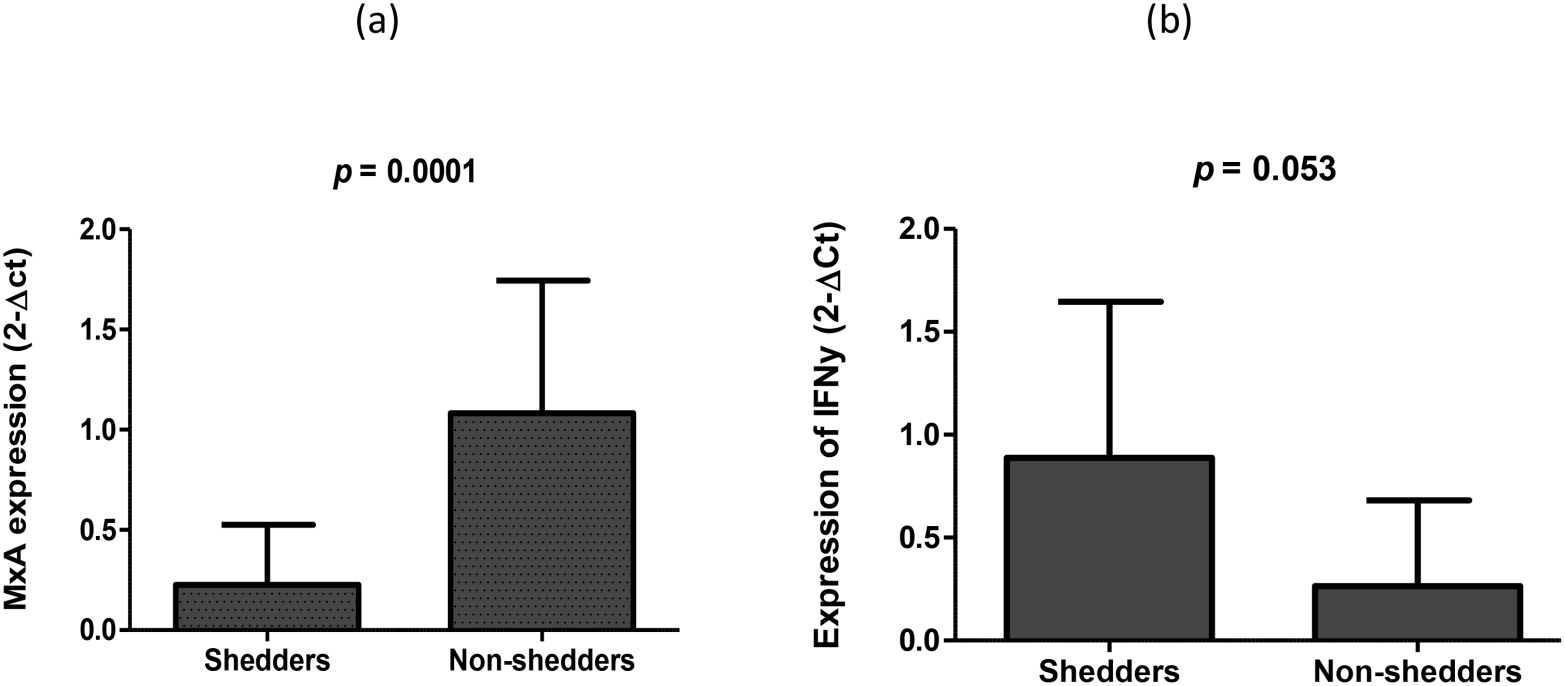
Average expression of **(a)** MxA (biomarker of IFN I and II secretion) and **(b)** IFN-γ 7 days after oral Rotarix vaccination between vaccine shedders and non-shedders.

We then measured the cytokine response following oral RV vaccination and observed that the average expression of MxA (IFN type 1) was increased 4.9-fold in non-shedders (2-ΔCt. 1.083) compared to vaccine shedders (2-ΔCt = 0.225), and the fold change in expression was statistically significant (*p* = 0.0001) (**Figure 3**). In contrast, the expression of IFN-γ was increased 3.06-fold in vaccine shedders (2-ΔCt = 0.0.807) compared to non-shedders (2-ΔCt = 0.263), *p* = 0.053.

### Association between MxA and Hepc expression and RV vaccine shedding

The expression of hepcidin, MxA (IFN I) or IFN-γ in saliva mRNA samples were used as continuous variables to evaluate their association with RV vaccine shedding in stool samples. Infants with elevated levels of hepcidin expression were less likely to shed the vaccine in stool samples (OR = 0.15, 95% CI = 0.002 – 10.49), but did not reach statistical significance, *p* = 0.136. Similarly, elevated levels of MxA expression was an impediment to RV vaccine virus replication as infants with higher levels of MxA expression were less likely to shed the vaccine virus in fecal samples [OR = 0.05, 95% CI = 0.008 – 0.296, *p* = 0.001). On the other hand, high levels of IFN-γ were predictive of RV vaccine shedding [OR = 18, 95% CI = 0.93 – 365], although it did not reach statistical significance, *p* = 0.056.

## Discussions

IDA, reported to be common in poor socio-economic settings (21), has been highlighted as one the factors affecting the performance of vaccines in low to middle income countries. Hepcidin is primarily produced in the liver and secreted in blood, and its levels in serum is used as an indicator of iron metabolism. However, the hormone has also been shown to be localized in the striated ducts of the sublingual and parotid glands, and salivary hepcidin concentration has been correlated with blood hepcidin levels (22). Recently, Guo et al. (23) observed that hepcidin can indeed be detected in the saliva, albeit in lower concentrations. In the present study, a higher percentage of infants maintained salivary hepcidin levels below the threshold level of 5.5 ng/ml pre- and post-vaccination, indicating that the frequency of iron deficiency anemia was very low in the study population. However, although the ELISA assay used in the study is specific and sensitive, it has a high possibility of false positive or negative results. Hence, we assessed whether the concentration of hepcidin was consistent with its expression in saliva, and observed that, unlike the concentration, the expression of salivary hepcidin was upregulated in non-shedders. This suggests that most of the infants in this study group were suffering from IDA i.e. were habitual iron blockers. Viruses require iron for replication and its unavailability may have prevented the replication of the vaccine virus, hence the observed poor vaccine shedding in stool samples.

There was a 2.4-fold decrease in hepcidin levels in post versus pre-vaccination in both vaccine shedders and non-shedders, suggesting a possible hepcidin suppressive dynamics either by the host or the virus itself. Some viral infections, such as Hepatitis C Virus (HCV) and Hepatitis B Virus (HBV), decrease hepcidin levels and boost iron absorption to promote their replication (12). A recent human study involving volunteers showed higher blood ferritin levels after HCV infection, which correlated with iron overload and inflammation (24), indicating an atypical drop in hepcidin levels. For HVC, the inhibition of hepcidin is regulated by the NS5A protein, which leads to an increase iron absorption (25). The function of HCV NS5A protein appears to be similar to the rotavirus NSP5, which Martin et al. (26) highlighted as a distinct viral metalloprotein that regulates iron during rotavirus replication in a cell to control its interaction with single-stranded RNA. Given the observed drop in hepcidin levels in the current study, we speculate that the live-attenuated G1[P8] strain contained in the vaccine downregulated hepcidin expression to boost enterocytes’ ability to absorb iron, allowing them to utilize iron to promote their replication and proliferation. Since iron is a necessary nutrient for the majority of viral infections, the rotavirus vaccine strain’s downregulation of hepcidin appears to be crucial for the vaccine take. It is also possible that other host hepcidin-suppressive dynamics were also at play - e.g., erythropoietic drive, and hypoxia (27, 28).

The recognition of viral pathogen-associated molecular patterns (PAMPS) is reported to induce inflammatory cytokines such as IL-6 (8), which in turn induces hepcidin expression (29) to limit the availability of iron in the cells. Hence, we assessed whether the expression of IL-6 was consistent with that of hepcidin between the two study groups, and found that, like hepcidin, it was upregulated in non-shedders compared to shedders. High levels of IL-6 have been reported in rotavirus infected individuals (30). However, it is not clear why the cytokine was upregulated only in non-shedders in the current study.

The viral nucleic acid is recognized as a PAMP during the early stages of the reproduction cycle of viruses within a cell. This recognition sets off a series of signaling processes that secretes IFNs crucial for antiviral immunity (31). Hence, we investigated the IFN I responses after the oral RV vaccination, and observed the downregulation of MxA in vaccine shedders versus non-shedders. MxA expression is a biomarker of secreted IFN I that is secreted in all nucleated cells exposed to viral infections (32). Our observation suggest that low levels of IFN I were secreted in shedders compared to non-shedders, and in absence of optimum antiviral immunity, the vaccine virus was able to proliferate, hence the shedding. This is consistent with a report that showed that the human wild-type strain Wa inhibits type I induced gene expression (low expression of MxA gene) via the degradation of IRF3 and inhibition of NF-κB activity that is mediated by the NSP1 (33). It is not clear why MxA was only upregulated in non-shedders but cannot exclude involvement of other factors including the indigenous microbiota that could have shaped the expression of MxA to calibrate the function of a broad range of immune cells, prompting them to respond and initiate adaptive immune responses when encountering pathogens (34). Gut microbiome, especially bacterial lipopolysaccharidesame and flagellin have previously been positively and negatively associated, respectively, with RV vaccine shedding in the same study population (35, 36).

IFN-γ has been shown to induce the expression of hepcidin (37) as cells treated with IFN-γ expressed high levels of hepcidin, suggesting that IFN-γ regulates the expression of hepcidin in humans. In another study, IFN-γ inhibited entry of RV into human intestinal cell lines, possibly by upregulating hepcidin (38). In contrast, IFN-γ was downregulated in non-shedders and upregulated in vaccine shedders in the current study. The differences could be attributed to the *in vitro* and *in vivo* settings used between the former and later studies. Nonetheless, our finding is consistent with observations made by Malil et al. (39), who found significant IFN-γ responses in children shedding with rotavirus candidate vaccine strain 116E. In the same study, the authors observed that NSP4 alone was able to induce significant IFN-γ response, suggesting that rotaviruses regulate IFN-γ responses through NSP4.

The study had limitations. Although hepcidin can be detected in saliva, its concentration is lower compared to serum. Further studies using serum samples are required to validate the current findings. We were also not able to measure the concentration of cytokines in saliva samples that would have validated their expression levels. There are also no standard cut-off values for distinguishing iron-absorbers (No IDA) from non-absorbers (IDA) as these values differ between different ELISA kits. Lastly, the number of samples used in the study was small and required cautious interpretation of the results.

In summary, we observed differences in salivary hepcidin concentration between vaccine shedders and non-shedders. However, its expression and that of hepcidin inducer IL-6 was much higher in non-shedders compared to shedders. In contrast, IFN-γ expression was increased significantly in vaccine shedders compared to non-shedders. Taken together, our findings suggests that IDA could impact RV vaccine take among South African infants. However, further studies using serum and large sample size are needed to validate the findings.

## Data Availability

The raw data supporting the conclusions of this article will be made available by the authors, without undue reservation.
